# Dibothriocephalosis in salmonids from Iceland: A more complex taxonomic problem than assumed until now?

**DOI:** 10.1016/j.crpvbd.2025.100314

**Published:** 2025-08-30

**Authors:** Lucia Dinisová, Eva Čisovská Bazsalovicsová, Karl Skírnisson, Ivica Králová-Hromadová

**Affiliations:** a Institute of Parasitology, Slovak Academy of Sciences, Hlinkova 3, 04001, Košice, Slovakia; b The University of Veterinary Medicine and Pharmacy in Košice, Komenského 68/73, 04181, Košice, Slovakia; c Institute for Experimental Pathology, University of Iceland, Keldnavegur 3, IS 112, Reykjavík, Iceland

**Keywords:** *Dibothriocephalus ditremus*, *Dibothriocephalus dendriticus*, *Diphyllobothrium hottai*, Mitochondrial haplotypes, Cytochrome *c* oxidase subunit 1, Genetic diversity

## Abstract

The diphyllobothriid tapeworm *Dibothriocephalus ditremus*, one of the three *Dibothriocephalus* species native to Europe, parasitises exclusively piscivorous birds and has not yet been detected in mammals. This is probably the reason why there is much less molecular data on this tapeworm. The aim of our study was to determine the genetic structure of the *D. ditremus* populations from Europe, namely Iceland, for the first time. To exclude any possible misidentifications between sympatrically occurring *D. ditremus* and *D. dendriticus*, *D. dendriticus* from Iceland was also analysed. Great genetic diversity of *D. ditremus*, displayed by a large number of cytochrome *c* oxidase subunit 1 (*cox*1) haplotypes and three distant clusters, contrasted sharply with the lower genetic variation in *D. dendriticus.* Previously published *cox*1 sequences of *D. ditremus* from different localities in Europe (UK - Scotland), Asia (Russia and Japan) and North America (USA - Oregon) were also included in the analysis in order to determine the genetic architecture of *D. ditremus* at a broader geographical scale. While the sequences of tapeworms from Scotland and Russia were placed in *D. ditremus* Clusters 2 and 3, the sample from USA (Oregon) displayed a unique position distant from the Icelandic tapeworms. Japanese samples of *D. ditremus* and *Diphyllobothrium hottai* formed a common clade, indicating their conspecificity. The unexpected output of the analysis was a unique position of the currently detected Haplotype 31 of a tapeworm from Iceland, which was distant from all other *D. ditremus* individuals from Iceland, but showed close relationships with the Japanese *D. ditremus*/*D. hottai* cluster. Further studies are needed to reveal if *D. ditremus* represent a complex of genetically diversified populations, or more species occur in salmonids in the Northern Hemisphere.

## Highlights

• Genetic structure of *Dibothriocephalus ditremus* populations from Iceland determined for the first time.
• Great genetic diversity of *D. ditremus* displayed by a large number of *cox*1 haplotypes.
• Three distinct *cox*1 clusters determined in *D. ditremus* from Iceland.
• Common clade of *D. ditremus* and *Diphyllobothrium hottai* from Japan indicating their conspecificity.
• One *D. ditremus* sample from Iceland closely related to Japanese *D. ditremus*/*D. hottai* cluster.

## Graphical abstract

**Figure:**
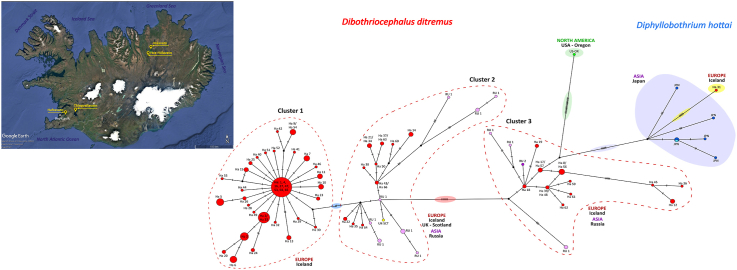

## 1 Introduction

Three species of *Dibothriocephalus* (Cestoda: Diphyllobothriidea), i.e. *Dibothriocephalus latus* (Linnaeus, 1758) (syn. *Diphyllobothrium latum*), *Dibothriocephalus dendriticus* (Nitzsch, 1824) (syn. *Diphyllobothrium dendriticum*) and *Dibothriocephalus ditremus* (Creplin, 1825) (syn. *Diphyllobothrium ditremum*), are native to Europe. While the most frequent definitive hosts of *D. latus* are humans, broader host specificity has been documented for *D. dendriticus*, which utilises a wide spectrum of definitive hosts including aquatic birds, mammals and occasionally humans (for a review see Kuchta et al., 2013, 2023; Králová-Hromadová et al., 2021, 2025a). As both species are the causative agents of dibothriocephalosis (fish-borne zoonosis), they have attracted the attention of parasitologists, as evidenced by several phylogenetic and genetic studies published in the last two decades (Wicht et al., 2010; Waeschenbach et al., 2017; Fraija-Fernández et al., 2021; Radačovská et al., 2022; Králová-Hromadová et al., 2025a).

*Dibothriocephalus ditremus*, on the other hand, parasitises piscivorous birds of the families Laridae (*Larus* spp. and *Rissa* spp.), Anatidae (*Aythya* spp., *Mergellus* spp., *Mergus* spp.), Gaviidae (*Gavia* spp.), Phalacrocoracidae (*Gulosus* spp. and *Phalacrocorax* spp.), Podicipedidae (*Podiceps* spp.) and Ardeidae (*Ardea* spp.) (e.g. Hickey and Harris, 1947; Vik, 1957, 1964; Halvorsen, 1970; Serdyukov, 1979; Sergeeva, 1983; Andersen et al., 1987; Dugarov et al., 2016) and has not been confirmed in mammals (Kuhlow, 1953; Halvorsen, 1970; Andersen, 1978). This is probably the reason why there is much less molecular and genetic data on this exclusively bird tapeworm.

Plerocercoids (larval stages in the second intermediate hosts) of *D. ditremus* are encapsulated in the body cavity on the internal organs of infected freshwater and anadromous fishes mainly of the family Salmonidae (e.g. *Coregonus* spp., *Salvelinus* spp., *Salmo* spp., *Oncorhynchus* spp., *Thymallus* spp.), but also of the families Gasterosteidae (*Gasterosteus* spp. and *Pungitius* spp.), Osmeridae (*Osmerus* spp. and *Hypomesus* spp.), Cottidae (*Cottocomephorus* spp. and *Cyphocottus* spp.) and Lotidae (*Lota* spp.) (Halvorsen, 1970; Bylund, 1975; Andersen et al., 1987; McDonald and Margolis, 1995; Pugachev, 2002; Banzai-Umehara et al., 2016; Henriksen et al., 2019).

The geographical distribution of *D. ditremus* is circumboreal, mainly in the Arctic and sub-Arctic zones of the Northern Hemisphere. Its occurrence in Europe is limited to Fennoscandia (Finland, Norway and Sweden) (Henricson, 1977; Tolonen and Kjellman, 2001; Henriksen et al., 2019), the Baltic region (Germany, Poland and Latvia) (Kuhlow, 1953; Rolbiecki et al., 1999; Kirjušina and Vismanis, 2007), the British Isles (Great Britain and Ireland) (Kennedy et al., 1991; Byrne et al., 2000), the North Atlantic islands (Iceland and Greenland) (Due and Curtis, 1995; Kristmundsson and Richter, 2009), and the Northwestern Federal District of the European part of Russia (Pugachev, 2002; Rumyantsev, 2007). In Asia, *D. ditremus* has been detected in the Asian part of Russia (Urals, Siberia and the Far East) (Pugachev, 2002) and in Japan (Banzai-Umehara et al., 2016), while in North America the species has been found in Canada and the USA (Andersen et al., 1987; McDonald and Margolis, 1995).

*Dibothriocephalus ditremus* was included in the phylogenetic study based on genes for the large and small subunits of nuclear rRNA (*lsr*DNA and *ssr*DNA), the large subunit of mitochondrial rRNA (*rrnL*) and the mitochondrial cytochrome *c* oxidase subunit 1 (*cox*1 mtDNA), which led to the resurrection of the genus *Dibothriocephalus* Lühe, 1899 from the polyphyletic genus *Diphyllobothrium* Cobbold, 1858 (Waeschenbach et al., 2017). The species was placed on an individual branch separated from the cluster including diphyllobothriids parasitising humans and/or mammals (*D. latus*, *D. dendriticus*, *Dibothriocephalus nihonkaiensis* and *Dibothriocephalus ursi*). The only population study on *D. ditremus* was published by Kutyrev and Mordvinov (2022), who investigated the genetic structure of *D. ditremus* and *D. dendriticus* from the Baikal Rift Zone in the Asian part of Russia. Comprehensive population genetic analyses of *D. ditremus* from Europe or North America have not yet been performed.

*Dibothriocephalus ditremus* occurs sympatrically and has a wide range of overlapping fish and bird hosts with *D. dendriticus* (see Supplementary Table S1 in Králová-Hromadová et al., 2025c). A recent study on the distribution, intensity of infection and prevalence of *D. dendriticus* and *D. ditremus* in salmonids from Iceland found that both tapeworms are present at a high prevalence in fishes from lakes across Iceland (Králová-Hromadová et al., 2025c). Additionally, a study on the genetic diversity and intercontinental dispersal of *D. dendriticus* populations from Europe, Asia, North America and South America revealed a mixed genetic architecture of *D. dendriticus* in Iceland, indicating multiple colonization events from the Nearctic and Palaearctic regions (Králová-Hromadová et al., 2025a). The intercontinental spread of *D. dendriticus* is ensured by aquatic migratory birds, which cross geographical barriers between continents. These results showed that Iceland occupies a strategic geographical position between Europe and North America and plays an important role in shaping the genetic structure of *Dibothriocephalus* tapeworms.

In contrast to *D. dendriticus*, studies on the genetic structure of *D. ditremus* have not yet been carried out in Europe. The present study aimed to determine the genetic structure of *D. ditremus* populations in Europe, namely in Iceland, for the first time. In order to exclude possible misidentifications between the sympatrically occurring *D. ditremus* and *D. dendriticus*, *D. dendriticus* obtained from the same localities and fish hosts as *D. ditremus* was also analysed. Previously published *cox*1 sequences of *D. ditremus* from different localities in Europe, Asia and North America were also included in the analyses to determine the genetic architecture of *D. ditremus* at a broader geographical scale.

## 2 Materials and methods

### 2.1 Parasite material

**Fig. 1:**
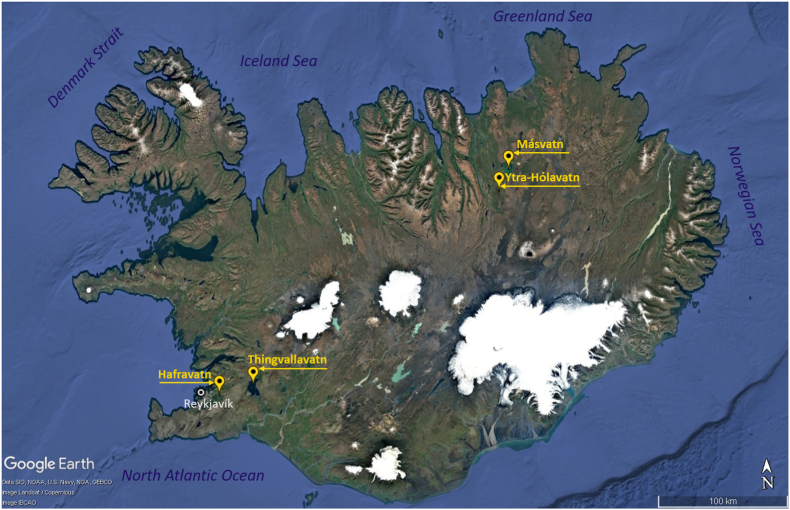
Scheme of the sampling sites of *Dibothriocephalus ditremus* and *Dibothriocephalus dendriticus* from Iceland studied in the present work. The basemap was obtained from Google Earth.

Plerocercoids were isolated from capsules localised in the abdominal cavity and on internal organs of infected brown trout (*Salmo trutta*) and Arctic charr (*Salvelinus alpinus*) from four lakes: Hafravatn (IS-HA) and Thingvallavatn (IS-TH), situated in the south-western part of the country, and Másvatn (IS-MA) and Ytra-Hólavatn (IS-YT), located in the north-eastern part of the island (Fig. 1). The fish were caught and euthanised by local professional fishermen. All larvae were dissected from the fish, rinsed in physiological saline solution and preserved in 96% ethanol for further analyses. A total of 192 plerocercoids of *D. ditremus* (IS-HA, *n* = 46; IS-TH, *n* = 47; IS-MA, *n* = 50; IS-YT, *n* = 49) and 132 larvae of *D. dendriticus* (IS-HA, *n* = 27; IS-TH, *n* = 51; IS-MA, *n* = 50; IS-YT, *n* = 4) were analysed (Table 1 and Supplementary Tables S1 and S2).

**Table 1:** Details on *Dibothriocephalus ditremus* and *Dibothriocephalus dendriticus* specimens ex *Salmo trutta* and *Salvelinus alpinus* from Iceland analysed in the present study.

Parasite species	Locality	Sample code	Fish host	*N*
*D. ditremus*	Hafravatn	IS-HA	*S*. *trutta*, *S*. *alpinus*	46
	Thingvallavatn	IS-TH	*S. alpinus*	47
	Másvatn	IS-MA	*S. trutta*	50
	Ytra-Hólavatn	IS-YT	*S. alpinus*	49
			Total	192
*D. dendriticus*	Hafravatn	IS-HA	*S. trutta*, *S. alpinus*	27
	Thingvallavatn	IS-TH	*S. alpinus*	51
	Másvatn	IS-MA	*S. trutta*	50
	Ytra-Hólavatn	IS-YT	*S. alpinus*	4
			Total	132

*Abbreviation*: *N*, number of samples.

Genomic DNA from individual larvae was isolated using the QIAamp® DNA Mini Kit (Qiagen, Hilden, Germany) according to the manufacturer’s recommendations. DNA was stored in deionised water at −20 °C. Species-specific PCR-based genotyping of all *Dibothriocephalus* spp. plerocercoids was performed with recently validated *Dibothriocephalus dendriticus*-specific forward Dd_8_F (5′-CGT CTA TGA TCA CGC ATG TCA-3′) and reverse Dd_8_R (5′-CGC TGT AGG ATT AGA TTC ACA CG-3′) primers (Králová-Hromadová et al., 2025b, 2025c). The PCR was performed in a total volume of 20 μl with 10–20 ng of genomic DNA, 10 pmol of each of the two primers and 1× PCR Master Mix (Thermo Fisher Scientific Inc., Waltham, USA). The PCR amplification conditions were 5 min at 95 °C as an initial denaturation step, followed by 40 cycles of 30 s at 95 °C, 1 min at 60 °C, 1 min at 72 °C, and a final polymerization step of 10 min at 72 °C.

### 2.2 Amplification, sequencing and sequence analysis of partial *cox*1

A partial mitochondrial *cox*1 fragment (891 bp) at the 3′-end of the gene was amplified for *D. ditremus* with forward DDI_COI_F (5′-GTG TTA GCT GCT GCT ATT AC-3′) and reverse DDI_COI_R (5′-TGA TAA GGA ACA GGA GCC C-3′) primers, and for *D. dendriticus* with forward DDE_COI_F (5′-CTT TTA CTT TTA ACT ATT CCT-3′) and reverse DDE_COI_R (5′-CTA TAA AGC CAA CAT ACT AT-3′) primers originally designed in the present study. PCR amplification was performed in a total volume of 20 μl with 10–20 ng of genomic DNA, 10 pmol of each of the two primers and 1× PCR Master Mix (Fermentas Life Sciences, Waltham, MA, USA). The PCR protocol for both species consisted of an initial denaturation step at 95 °C for 5 min, followed by 29 cycles of denaturation at 95 °C for 1 min, annealing at 55 °C for 1 min for *D. ditremus* or at 50 °C for 1 min for *D. dendriticus* and extension at 72 °C for 2 min. The PCR products were visualised on 1% agarose gel and purified using an ExoProStar™ 1-STEP Kit (Illustra, Chicago, IL, USA). Each PCR product for *D. ditremus* and *D. dendriticus* was sequenced from both sides using primers applied for PCR amplification. Sequencing was performed using an Automatic Genetic Analyser 3130xl and BigDye Terminator v.3.1 Cycle sequencing kit (Applied Biosystems, Foster City, CA, USA). The chromatograms of the sequences were manually trimmed and assembled using Geneious software (version 10.0.5, Biomatters, Auckland, New Zealand). Two independent sets of raw sequence data were checked and aligned to obtain the final contiguous sequences. The newly generated *cox*1 sequences of *D. ditremus* from Iceland were deposited in the GenBank database under the accession numbers PV928216-PV928258, PV928261-PV928278, PV928300-PV928309, PV928537-PV928545, PV928668-PV928675, PV928715-PV928721, PV928743-PV928747, PV928943-PV928958, PV928992-PV929006, PV929008-PV929029, PV929048-PV929086 (see Supplementary Table S1 for details). The newly generated *cox*1 sequences of *D. dendriticus* from Iceland were deposited in the GenBank database under the accession numbers PV918954-PV918990, PV919004-PV919034, PV919038-PV919062, PV919063-PV919071, PV919135-PV919142, PV919149-PV919153, PV919461-PV919463, PV919473-PV919475, PV919490-PV919492, PV919509-PV919510, PV919515-PV919520 (see Supplementary Table S2 for details).

### 2.3 Haplotype networks

Genealogical information of *D. ditremus* and *D. dendriticus* populations was visualised *via* a partial *cox*1 haplotype network (891 bp) using PopArt (Leigh and Bryant, 2015) with the TCS 1.21 algorithm (Clement et al., 2000). The haplotype networks were displayed independently for the currently analysed *D. ditremus* (*n* = 192) and *D. dendriticus* (*n* = 132) from Iceland. Both species were also analysed together in the common analysis (*n* = 324). Additionally, the *cox*1 sequences of *D. ditremus* from Iceland were analysed together with the GenBank data of *D. ditremus* from Europe (UK - Scotland, *n* = 1; Wicht et al., 2010), Asia (Japan, *n* = 1; Banzai-Umehara et al., 2016), Russia (*n* = 17; Kutyrev and Mordvinov, 2022) and North America (USA - Oregon, *n* = 1; Waeschenbach et al., 2017) (Table 2). Since the comparative *cox*1 sequences were available either as complete (1566 bp) or partial (678 bp) sequences, they were all trimmed to a comparable length of 678 bp. In addition to *D. ditremus*, *Diphyllobothrium hottai* Yazaki, Fukumoto & Abe, 1988, a genetically similar (or identical) species to *D. ditremus* from Japan, was also included in the analysis (*n* = 6; Banzai-Umehara et al., 2016).

**Table 2:** Summary of comparative *cox*1 sequence data of *Dibothriocephalus ditremus* and *Diphyllobothrium hottai* from different continents/countries.

Continent	Country	Locality	Fish host	GenBank ID	*N*	Length (bp)	Reference
***Dibothriocephalus ditremus***							
Europe	UK (Scotland)	Loch Doyne	*Salvelinus alpinus*	FM209182	1	1566^a^	Wicht et al. (2010)
North America	USA (Oregon)	McKenzie River	*Oncorhynchus tshawytscha*	KY552872	1	1566^a^	Waeschenbach et al. (2017)
Asia	Japan	Hokkaido	*Hypomesus pretiosus japonicus*	AB979518	1	1566^a^	Banzai-Umehara et al. (2016)
Asia	Russia	Lake Baikal	*Coregonus migratorius*	MW979733	1	678^b^	Kutyrev and Mordvinov (2022)
Asia	Russia	Lake Kapylushi	*Coregonus baunti*	MW979734-MW979749	16	678^b^	Kutyrev and Mordvinov (2022)
***Diphyllobothrium hottai***							
Asia	Japan	Hokkaido	*H. pretiosus japonicus*	AB979516, AB979517, AB979519-AB979522	6	1566^a^	Banzai-Umehara et al. (2016)

*Abbreviations*: *N*, number of sequences.

a Complete *cox*1 sequence.

b *cox*1 sequences shorter than 678 bp were not included in the analysis.

### 2.4 Statistics

The programme DnaSP 6 (Rozas et al., 2017) was used to estimate the number of segregating sites, the number of parsimony-informative sites, genetic diversity, haplotype diversity (*Hd*), nucleotide diversity (*π*), and neutrality test statistics (Fu and Li’s *F*∗, Tajima’s *D*, Ramos-Onsins and Rozas’ *R*_*2*_). The significance of all tests was determined by 10,000 coalescent simulations. The statistical parameters were calculated independently for the newly analysed populations of *D. ditremus* and *D. dendriticus* from Iceland.

## 3 Results

### 3.1 Structure and diversity of *cox*1 haplotypes

#### 3.1.1 Dibothriocephalus ditremus

The mitochondrial *cox*1 sequences of 192 individuals of *D. ditremus* from Iceland (see GenBank accession numbers in Supplementary Table S1) were sorted into 66 haplotypes (Ddi_CO1-Ha1 to Ha66) (Supplementary Table S3). Most haplotypes differed from the reference Haplotype 1 (Ddi_CO1-Ha1) by 1–8 mutations, but 15 haplotypes (Haplotypes 8, 12, 17, 18, 19, 31, 36, 39, 45, 48, 56, 57, 59, 61, and 62) differed substantially from Haplotype 1 by 25–28 mutations (Supplementary Table S3, haplotypes highlighted in yellow) and Haplotype 31 even by 34 mutations (Supplementary Table S3, haplotype highlighted in blue). Of 103 mutations, 92 transitions and 11 transversions were detected in the entire data set; 91 mutations were synonymous and 12 were nonsynonymous, altering the protein amino acid sequence (Supplementary Table S3).

**Fig. 2:**
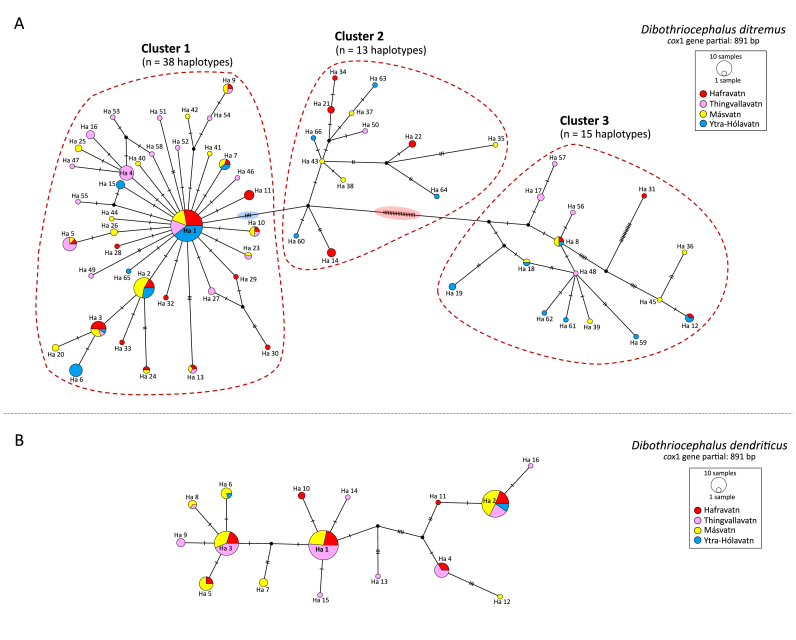
Haplotype network based on the mitochondrial *cox*1 haplotypes of *Dibothriocephalus ditremus* (**A**) and *Dibothriocephalus dendriticus* (**B**) from Iceland. Circle size is proportional to the number of samples; each hatch mark represents a single mutation; a black dot symbolises an intermediate missing or unsampled haplotype. Details of the haplotypes are presented in Supplementary Tables S1 and S2.

The haplotype network of *D. ditremus* showed three clearly distinguishable clusters (Fig. 2A). Cluster 1 was represented by the highest number of haplotypes (*n* = 38), which were detected in 152 individuals. This cluster showed a typical star-like pattern with the most numerous central haplotype, Haplotype 1, which was detected in 43 *D. ditremus* individuals from all four lakes. The second most numerous Haplotype 2 was determined in 18 individuals from three lakes (Hafravatn, Másvatn, and Ytra-Hólavatn). Thirty-seven haplotypes were placed around the central Haplotype 1 on individual mutational pathways separated from Haplotype 1 by 1–6 mutations.

Cluster 2 included 13 haplotypes detected in 17 individuals and was displayed by a tree-like pattern. It was separated from Cluster 1 by four mutations (Fig. 2A, see mutations highlighted in blue). The haplotypes within Cluster 2 were detected in a smaller number of individuals (*n* = 1–3), and each of them was detected only in one locality.

Cluster 3 was visualised as a diffuse network of 15 haplotypes detected in 23 individuals and was placed on a distant mutational pathway separated from Cluster 2 by 20 mutations (Fig. 2A, see mutations highlighted in red). Cluster 3 included distinct haplotypes that differed from Haplotype 1 by 25–34 mutations (Supplementary Table S3). The haplotypes in Cluster 3 were mainly detected in 1–2 individuals, and the most numerous haplotypes, Haplotype 8 and Haplotype 12, were found in four individuals from three (Hafravatn, Másvatn, and Ytra-Hólavatn) and two (Hafravatn and Ytra-Hólavatn) lakes, respectively.

No genetic structuring of *D. ditremus* haplotypes associated with the respective locality was detected (Fig. 2A). The haplotypes of *D. ditremus* displayed 98.8–99.9% and 97.6–99.9% sequence identity within Clusters 1+2 and Cluster 3, respectively. Lower values (95.8–97.3%) were detected between *D. ditremus* clusters 1+2 and Cluster 3 (Table 3).

**Table 3:** Percentage identity (%) between the partial mitochondrial *cox*1 (891 bp) of *Dibothriocephalus ditremus* and *Dibothriocephalus dendriticus* from Iceland and *Diphyllobothrium hottai* from Japan.

	*D. ditremus* Clusters 1+2	*D. ditremus* Cluster 3	*D. dendriticus*	*D. hottai*
*D. ditremus* Clusters 1+2	98.8–99.9			
*D. ditremus* Cluster 3	95.8–97.3	97.6–99.9		
*D. dendriticus*	89.1–90.1	89.0–90.7	98.8–99.9	
*D. hottai*	95.6–96.5	97.9–99.1	89.1–89.9	99.1–99.9

#### 3.1.2 Dibothriocephalus dendriticus

Substantially lower haplotype diversity was detected in *D. dendriticus*, in which 16 *cox*1 haplotypes (Dde_CO1-Ha1 to Ha16) were identified in 132 specimens (Supplementary Table S4); their GenBank accession numbers are presented in Supplementary Table S2. Of 25 mutations, the transitions to transversions ratio was 24:1 and the ratio of synonymous to nonsynonymous mutations was 21:4 (Supplementary Table S4).

The haplotype network of *D. dendriticus* had a linear pattern and was manifested by three dominant haplotypes: Haplotypes 1, 2 and 3 (Fig. 2B). Haplotype 1 and Haplotype 3 were detected in 37 and 25 individuals, respectively, from three lakes (Hafravatn, Thingvallavatn, and Másvatn), while Haplotype 2 was determined in 31 specimens from all four localities. Haplotypes 2 and 6 were the only haplotypes detected in samples from Ytra-Hólavatn, which can be explained by a much smaller number of specimens from this lake that were available for the analysis. No genetic structuring of *D. dendriticus* haplotypes associated with the respective locality was detected (Fig. 2B). The sequence identity among *D. dendriticus* haplotypes was 98.8–99.9% (Table 3).

#### 3.1.3 Dibothriocephalus ditremus versus Dibothriocephalus dendriticus

**Fig. 3:**
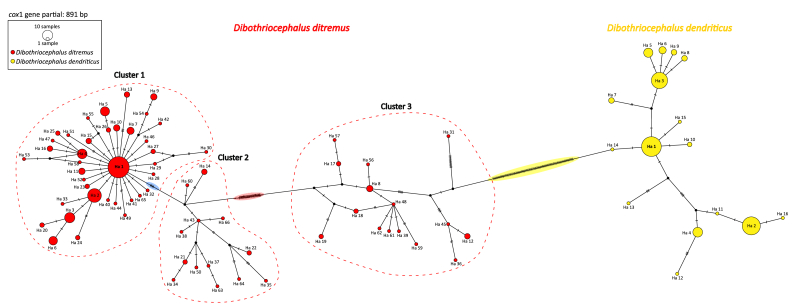
Common haplotype network based on the mitochondrial *cox*1 haplotypes of *Dibothriocephalus ditremus* and *Dibothriocephalus dendriticus* from Iceland. Circle size is proportional to the number of samples; each hatch mark represents a single mutation; a black dot symbolizes an intermediate missing or unsampled haplotype.

Given the markedly greater genetic diversity observed in *D. ditremus* compared to *D. dendriticus*, a potential misidentification and overlap in their haplotype networks, mainly between the distant *D. ditremus* Cluster 3 and *D. dendriticus*, had to be ruled out. The common haplotype network (891 bp) of all analysed *D. ditremus* (192 samples; 66 haplotypes) and *D. dendriticus* individuals (132 samples; 16 haplotypes) from Iceland showed two clearly defined and distinct haplotype networks specific to each species (Fig. 3). The most distant Cluster 3 of *D. ditremus* haplotypes was separated from *D. dendriticus* by 77 mutations (Fig. 3, see mutations highlighted in yellow), indicating a substantially greater genetic distance than between Clusters 1+2 and Cluster 3 of *D. ditremus* haplotypes (20 mutations; Fig. 3, see mutations highlighted in red).

The interspecific differences between both species were displayed by lower values of sequence identity between *D. dendriticus* and *D. ditremus* Clusters 1+2 (89.1–90.1%), and *D. dendriticus* and *D. ditremus* Cluster 3 (89.0–90.7%) (Table 3). To conclude, *D. ditremus* Cluster 3, despite being rather distant from *D. ditremus* Clusters 1+2, was genetically closer to *D. ditremus* than to *D. dendriticus.*

### 3.2 Haplotype network of present *D. ditremus* data and GenBank data

**Fig. 4:**
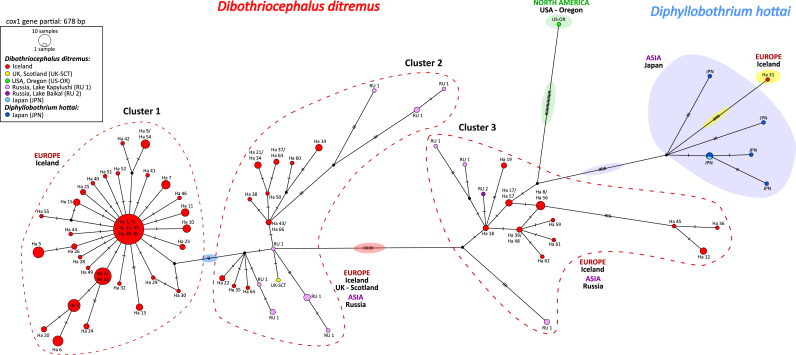
Haplotype network based on the mitochondrial *cox*1 haplotypes of *Dibothriocephalus ditremus* from Iceland (original data) and previously published *D. ditremus* from UK (Scotland), Russia, USA (Oregon), Japan, and *Diphyllobothrium hottai* from Japan (data from GenBank; see Table 2 for details). Circle size is proportional to the number of samples; each hatch mark represents a single mutation; a black dot symbolizes intermediate missing or unsampled haplotype.

The haplotype network based on *cox*1 sequences of presently analysed *D. ditremus* from Iceland along with GenBank data for *D. ditremus* from Europe (UK - Scotland), Asia (Russia and Japan), North America (USA - Oregon) and *D. hottai* from Japan is shown in Fig. 4. None of the comparative sequences were included in *D. ditremus* Cluster 1, which was specific to Iceland. A sequence of a tapeworm from Scotland was only placed in Cluster 2 on separate mutational pathways, while sequences of *D. ditremus* from the Asian part of Russia were assigned to Clusters 2 and 3. One sample from North America (USA - Oregon) displayed a unique position rather distant from *D. ditremus* Clusters 1+2 and separated from *D. ditremus* Cluster 3 by 19 mutations (Fig. 4, see mutations highlighted in green).

The Japanese samples of *D. ditremus* and *D. hottai* formed a common cluster, indicating their conspecificity. This cluster was distant from *D. ditremus* Clusters 1+2, but close to *D. ditremus* Cluster 3, from which it was separated by six mutations (Fig. 4, see mutations highlighted in violet).

The unexpected output of this analysis was a unique position of Haplotype 31 of a diphyllobothriid tapeworm from Iceland, which was distant from all other tapeworms from Iceland, suggesting its different taxonomy. The sample showed close relationships to the Japanese *D. ditremus*/*D. hottai* cluster, from which it was separated by six mutations (Fig. 4, see mutations highlighted in yellow).

The sequence identity between *D. hottai* and *D. dendriticus* was 89.1–89.9% and corresponded to the interspecific differences between *D. dendriticus* and *D. ditremus* (89.0–90.7%) (Table 3). However, the sequence identity between *D. hottai* and *D. ditremus* was 95.6–96.5% (*D. hottai* and *D. ditremus* Clusters 1+2) and 97.9–99.1% (*D. hottai* and *D. ditremus* Cluster 3); these values did not correspond to the interspecific differences, but they reflected the values of intraspecific variability (Table 3). A particularly high genetic similarity was determined between *D. hottai* and *D. ditremus* Cluster 3.

### 3.3 Statistical analyses of the *cox*1 data

The results from statistical analysis of the genetic diversity of *D. ditremus* and *D. dendriticus* from Iceland are presented in Table 4. The entire dataset of *D. ditremus* (192 individuals) contained 98 segregating sites, of which 61 were parsimony informative. In contrast, *D. dendriticus* data set (132 individuals) contained 25 segregating sites, of which 17 were parsimony informative. The level of haplotype diversity was comparable in the entire data sets and individual populations of both species, except for *D. dendriticus* from Ytra-Hólavatn, which showed the lowest haplotype diversity (0.500), apparently due to the low number of tapeworms analysed. The nucleotide diversity was lower in the *D. dendriticus* populations compared to that in *D. ditremus*.

**Table 4:** Molecular variability and tests of neutrality of mitochondrial haplotypes for different populations of *Dibothriocephalus ditremus* and *Dibothriocephalus dendriticus* from Iceland.

Lake	*N*	S	PIS	*n*	*Hd*	*π*	Fu and Li’s *F*∗	Tajima’s *D*	Ramos-Onsins & Rozas’ *R*_*2*_
***Dibothriocephalus ditremus***									
Entire data set	192	98	61	66	0.932	0.00940	−3.0611 (*P =* 0.00460)∗∗	−1.6352 (*P* = 0.01800)∗∗	0.0415 (*P* = 0.0348)∗
Hafravatn	46	64	39	22	0.913	0.00797	−1.7247 (*P* = 0.06290)	−1.8915 (*P* = 0.00940)∗∗	0.0526 (*P* = 0.01350)∗∗
Thingvallavatn	47	53	34	24	0.929	0.00834	−1.4153 (*P* = 0.09410)	−1.4102 (*P* = 0.06050)	0.0653 (*P* = 0.05360)
Másvatn	50	58	42	26	0.938	0.00939	−0.8889 (*P* = 0.19020)	−1.3090 (*P* = 0.07570)	0.0675 (*P* = 0.09030)
Ytra-Hólavatn	49	50	40	18	0.853	0.01144	−0.0261 (*P* = 0.49060)	−0.4365 (*P* = 0.37230)	0.0981 (*P* = 0.42730)
***Dibothriocephalus dendriticus***									
Entire data set	132	25	17	16	0.825	0.00510	−1.1419 (*P* = 0.13890)	−0.1367 (*P* = 0.51820)	0.0895 (*P* = 0.55890)
Hafravatn	27	12	12	7	0.835	0.00501	1.5809 (*P* = 0.97140)	1.4521 (*P* = 0.94460)	0.1861 (*P* = 0.95990)
Thingvallavatn	51	18	11	10	0.792	0.00453	−0.8535 (*P* = 0.20180)	0.0265 (*P* = 0.56980)	0.1079 (*P =* 0.55840)
Másvatn	50	17	14	8	0.828	0.00549	0.4921 (*P =* 0.69870)	0.6829 (*P =* 0.79880)	0.1430 (*P =* 0.87510)
Ytra-Hólavatn	4	10	0	2	0.500	0.00561	–	–	–

*Abbreviations*: *N*, number of analysed samples; S, number of segregating sites; PIS, number of parsimony-informative sites; *n*, number of haplotypes; *Hd*, haplotype diversity; *π*, nucleotide diversity; –, data not calculated.

*Note*: Levels of significance: ∗*P* < 0.05; ∗∗*P* < 0.02.

Neutrality tests were applied to: (i) the entire data sets and (ii) individual populations for each species in order to elucidate past demographic events. For *D. ditremus*, the values of Fu and Li’s *F*∗ and Tajima’s *D* showed a significant deviation from neutrality and expected equilibrium in the entire data set (−3.0611, *P* = 0.00460 and −1.6352, *P* = 0.01800, respectively) and in the population from Hafravatn (−1.8915, *P* = 0.00940). Ramos-Onsins and Rozas’s *R*_*2*_ yielded significant positive values also in the entire dataset (0.0415, *P* = 0.0348) and in the population from Hafravatn (0.0526, *P* = 0.01350). The combination of significantly negative Fu and Li’s *F*∗ and Tajima’s *D* provides evidence for an excess of low-frequency variants, consistent with either recent population expansion or purifying selection. A low and significant *R*_*2*_ value supports the hypothesis of recent population growth.

For *D. dendriticus*, the neutrality tests for the entire dataset yielded non-significant results in all three statistics. The values of Fu and Li’s *F*∗ (−1.1419, *P* = 0.13890) and Tajima’s *D* (−0.1367, *P* = 0.51820) were both negative, indicating a mild excess of rare variants. The lack of statistical significance indicates no deviation from neutrality. Ramos-Onsins and Rozas’s *R*_*2*_ (0.0895, *P* = 0.55890) also failed to reject neutrality.

The non-significant results of the neutrality tests for the populations sampled in Hafravatn, Másvatn, and Thingvallavatn, indicate that the data are consistent with neutral evolution and demographic stability. Ramos-Onsins and Rozas’s *R*_*2*_ yielded non-significant positive values, largely indicative of neutral selection or populations evolving as per the mutation-drift equilibrium. Statistical parameters could not be calculated for the Ytra-Hólavatn population due to small sample size.

## 4 Discussion

The present study revealed a great genetic diversity in *D. ditremus*, evidenced by a large number of *cox*1 haplotypes and three distant clusters, in sharp contrast to lower genetic variation in *D. dendriticus*, presented by a smaller number of haplotypes and a much simpler haplotype network. Such genetic differences between these two species of *Dibothriocephalus* were not anticipated because both species share several biological characteristics, including a similar life cycle and many common fish and bird hosts (see Supplementary Table S1 in Králová-Hromadová et al., 2025c).

Migratory aquatic birds, the only definitive hosts of *D. ditremus* and the most common hosts of *D. dendriticus*, play a key role in shaping the genetic structure of both tapeworm species, as the high mobility of birds helps to spread the eggs not only in Iceland but also over long distances and even between continents. Iceland is situated along the East Atlantic Flyway, which connects North America (including Greenland and eastern Canada) with Europe (Caliendo et al., 2022). As for *D. dendriticus*, the most frequent definitive bird hosts are gull species breeding or wintering in Iceland, Greenland, Canada, the British Isles and the Scandinavian Atlantic coast, and their regular seasonal migrations have apparently resulted in transmission of eggs of *D. dendriticus* throughout the northern Palaearctic and Nearctic (Králová-Hromadová et al., 2025a). On the other hand, *D. ditremus* is more frequently linked with divers, goosanders and mergansers (Borgstrøm et al., 2017), which also migrate among Iceland, Europe and North America (Spina et al., 2022; BirdLife, 2025).

The only pronounced difference between *D. ditremus* and *D. dendriticus* is the intensity of infection of salmonids, which has recently been studied in detail in lakes Hafravatn, Thingvallavatn, Másvatn and Ytra-Hólavatn in Iceland (Králová-Hromadová et al., 2025c). The intensity of infection with *D. dendriticus* was lower in all four lakes than the higher levels observed for *D. ditremus*. This phenomenon has been previously observed in Norway and Sweden and was explained by the different climatic conditions required for the development of the eggs of both species and the different feeding ecology of aquatic birds, mainly gulls (*D. dendriticus*) and divers, goosanders, and mergansers (*D. ditremus*) (Halvorsen, 1970; Halvorsen and Wissler, 1973; Henricson, 1977). It has been shown that the eggs of *D. ditremus* can survive in near-Arctic conditions (Vik, 1957; Halvorsen, 1970), such as in Svalbard, one of the world’s northernmost archipelagos located in the Arctic Circle (Hammar, 2000).

The higher genetic diversity of tapeworms may be due to various factors, such as reproductive modes, evolutionary history, seasonal and annual fluctuations and different ecosystems (Yamasaki et al., 2023). Another parameter that effects high genetic diversity is the size of the population, as the most abundant species tends to be genetically most diverse (Nabholz et al., 2008). Accordingly, the higher intensity of infection of *D. ditremus* in Iceland (Králová-Hromadová et al., 2025c) could explain its great genetic diversity detected in the present study.

The present data can be compared with a previously published genetic structure of *D. ditremus* and *D. dendriticus* from the Baikal Rift Zone in the Asian part of Russia (Kutyrev and Mordvinov, 2022). The phylogenetic analyses and *cox*1 haplotype networks showed two distinct clades for both species, with genetic patterns within species not so markedly different in comparison to distinct patterns observed for both species in Iceland. In the Baikal Rift Zone, haplotype diversity was slightly higher in *D. ditremus* than in *D. dendriticus*, while nucleotide diversity was significantly higher in *D. dendriticus* (Kutyrev and Mordvinov 2022). In Iceland, *D. ditremus* showed both higher haplotype and nucleotide diversity.

The taxonomy and biology of *Dibothriocephalus* (formerly *Diphyllobothrium*) tapeworms were rather confusing in the mid-20th Century, when seven species were supposed to occur in northern and western Europe (Halvorsen, 1970), i.e. *D. latum*, *D. dendriticum*, *D. ditremum*, *Diphyllobothrium osmeri* (Linstow, 1878), *Diphyllobothrium vogeli* Kuhlow (1953), *Diphyllobothrium norvegicum* Vik (1957) and *Diphyllobothrium medium* (Fahmy, 1954). According to results based on natural and experimental infections conducted in the 1960’s, *D. osmeri* and *D. vogeli* were synonymised with *D. ditremum*, while *D. norvegicum* and *D. medium* were synonymised with *D. dendriticum* (Chubb, 1968). Finally, only three species, *D. latum*, *D. dendriticum* and *D. ditremum*, were considered as valid in Europe (Chubb, 1968). However, the taxonomy at that time could rely solely on morphology, which is rather complicated in the order Diphyllobothriidea due to the high intraspecific morphological variability, wide range of intermediate and definitive hosts and morphological artefacts caused by fixation (Andersen et al., 1987). As the European populations of *D. ditremus* have not been investigated using DNA-based methods, the results published by Chubb (1968) more than 50 years ago were considered reliable until today. However, it appears that the indication for several species of *Dibothriocephalus* parasitising salmonids in northern Europe, as proposed by the traditional “Old School” taxonomists in the middle of the last century, was probably correct.

Another explanation for the present results is that a complex of genetically different *D. ditremus* populations circulates in Iceland and also in the other regions where this tapeworm is found in the Northern Hemisphere. The most striking differences observed in the present study were: (i) the closer position of the Icelandic *D. ditremus* Cluster 3 to the Japanese *D. ditremus/D. hottai* cluster; (ii) the distant position of a single tapeworm from Iceland (IS-HA/5/2; Haplotype 31) from all three *D. ditremus* clusters from Iceland and its close affiliation to the Japanese *D. hottai/D. ditremus* cluster.

The validity of *D. hottai* in Japan has been the subject of debate and is obviously still an unresolved issue. The first data on *D. hottai* were published by Hotta et al. (1978), who described several plerocercoids from the Japanese surf smelt (*Hypomesus pretiosus japonicus*) and the olive rainbow smelt (*Osmerus eperlanus mordax*) from Hokkaido. The authors performed experimental infections of golden hamsters in which adult tapeworms reached maturity. Even though the adult tapeworms were morphologically very similar to *D. ditremus*, further studies on morphology, biology and isozyme patterns (Yazaki et al., 1986; Fukumoto et al., 1987) led to the description of a new species, *Diphyllobothrium hottai* Yazaki, Fukumoto & Abe, 1988 (Yazaki et al., 1988).

Two decades later, a single plerocercoid isolated from the Japanese smelt was molecularly identified using the rRNA gene region (1238 bp) and partial *cox*1 (428 bp), which showed 98.3% and 98.5% identity to *D. ditremus*, respectively (Abe, 2009). The author concluded, that *D. hottai* and *D. ditremus* represent a single species and questioned the validity of *D. hottai*.

Similar conclusions were reached by Banzai-Umehara et al. (2016). The authors analysed *D. hottai* and *D. ditremus* obtained from hamsters experimentally infected with plerocercoids isolated from the Japanese surf smelts. Phylogenetic analysis of six adult worms of *D. hottai* and one of *D. ditremus* based on the complete *cox*1 and *cob* sequences revealed that the two species were genetically indistinguishable as they clustered together. Banzai-Umehara et al. (2016) concluded that *D. hottai* is a junior synonym of *D. ditremus*.

To make the story even more complicated, Nakao and Yanagida (2018) claimed that the detection of *D. ditremus* in Japan was probably a misidentification, and only *D. hottai* is present in Japan. It is worth noting that *D. ditremus* prefers freshwater fishes while *D. hottai* is associated with marine smelts as second intermediate hosts. Nakao and Yanagida (2018) proposed further comparative analyses based on broader sampling of *D. ditremus* from Europe and *D. hottai* from Japan, which would clarify the taxonomic status of the *D. ditremus* complex.

All of the above-mentioned studies agreed on the great genetic similarity between *D. ditremus* and *D. hottai*, suggesting the presence of only *D. ditremus* (Abe, 2009; Banzai-Umehara et al., 2016) or only *D. hottai* (Nakao and Yanagida, 2018) in Japan. Our results confirmed that the Japanese *D. ditremus* and *D. hottai* belong to the same species, as they clustered together. However, we do not have sufficient data to decide whether the Japanese tapeworms belong to *D. ditremus* or *D. hottai* and, consequently, if the distant tapeworm found in Iceland is *D. hottai*. Under the given circumstances, we fully agree with the conclusions of Blair (2018) that populations on the edge of speciation and geographically separated populations can be difficult to characterise within the binomial system. Until sufficient biological and genetic data are obtained for clarification, the best solution is to regard *D. ditremus* and *D. hottai* as members of a species complex (Blair, 2018).

Similar to the samples from Japan, *D. ditremus* from the USA (Oregon) was found to have a distant genetic structure and/or questionable taxonomy. Further studies based on a complex multidisciplinary approach, including detailed morphological and molecular data, are required to confirm whether the tapeworms from Japan and Oregon represent genetically distant populations of *D. ditremus* or new species, genetically closely related to *D. ditremus*.

## 5 Conclusions

The first findings on the extreme intraspecific polymorphism of *D. ditremus* have opened a challenging new chapter in the study of diphyllobothriids parasitising salmonids in northern Europe. Geographically broader sampling of *D. ditremus* populations from north-west Europe (the British Isles and Fennoscandia), North America (Canada and USA) and Asia (Russia and Japan) needs to be conducted, and samples need to be investigated in detail to clarify if *D. ditremus* represents a genetically diverse species complex or whether more species of *Dibothriocephalus/Diphyllobothrium* circulate in salmonids in the Northern Hemisphere. A greater number of individuals from a wide range of geographical areas will enable a more accurate assessment of population-level genetic diversity and global biogeographical patterns of *D. ditremus*. Future studies may shed light on whether the Icelandic tapeworm, for which the distant Haplotype 31 was identified, represents a species of *Dibothriocephalus/Diphyllobothrium* previously undiscovered in Europe and if this species was “a lost intercontinental traveller” or “a permanent resident of Iceland”.

## Ethical approval

Not applicable.

## CRediT authorship contribution statement

**Lucia Dinisová:** Formal analysis, Investigation, Methodology, Validation, Visualization, Writing – review & editing. **Eva Čisovská Bazsalovicsová:** Formal analysis, Methodology, Validation, Supervision, Writing – review & editing. **Karl Skírnisson:** Resources, Writing – review & editing. **Ivica Králová-Hromadová:** Conceptualization, Funding acquisition, Project administration, Supervision, Writing – original draft, Writing – review & editing.

## Funding

This work was financially supported by the 10.13039/501100005357Slovak Research and Development Agency (project no. APVV-23-0390).

## Declaration of competing interests

The authors declare that they have no known competing financial interests or personal relationships that could have appeared to influence the work reported in this paper.

## Data Availability

The data supporting the conclusions of this article are included within the article and its supplementary files. The newly generated *cox*1 sequences of *D. ditremus* were deposited in the GenBank database under the accession numbers PV928216-PV928258, PV928261-PV928278, PV928300-PV928309, PV928537-PV928545, PV928668-PV928675, PV928715-PV928721, PV928743-PV928747, PV928943-PV928958, PV928992-PV929006, PV929008-PV929029, PV929048-PV929086. The newly generated *cox*1 sequences of *D. dendriticus* were deposited in the GenBank database under the accession numbers PV918954-PV918990, PV919004-PV919034, PV919038-PV919062, PV919063-PV919071, PV919135-PV919142, PV919149-PV919153, PV919461-PV919463, PV919473-PV919475, PV919490-PV919492, PV919509-PV919510, PV919515-PV919520.
