# Supplementary material for: Dibothriocephalosis in salmonids from Iceland: A more complex taxonomic problem than assumed until now?
Source: Curr Res Parasitol Vector Borne Dis. 2025 Aug 30;8:100314. doi: 10.1016/j.crpvbd.2025.100314 (PMC12863047; doi:10.1016/j.crpvbd.2025.100314)
Supplement: Multimedia component 1 [file mmc1.pdf]

Supplementary Table S1. Summary of *Dibothriocephalus ditremus* specimens from Iceland analysed in the present study and details of mitochondrial cytochrome *c* oxidase subunit 1 haplotypes (Ddi_CO1-Ha; 891 bp).

Lake	Host	Ddi_CO1-Ha	GenBank Acc. No.
**HAFRAVATN (IS-HA)**			
IS-HA/1/7	*Salmo trutta*	Ddi_CO1-Ha2	PV928261
IS-HA/1/9	*S. trutta*	Ddi_CO1-Ha2	PV928262
IS-HA/2/9	*S. trutta*	Ddi_CO1-Ha21	PV929016
IS-HA/2/11	*S. trutta*	Ddi_CO1-Ha1	PV928216
IS-HA/2/13	*S. trutta*	Ddi_CO1-Ha7	PV928743
IS-HA/3/2	*S. trutta*	Ddi_CO1-Ha1	PV928217
IS-HA/3/4	*S. trutta*	Ddi_CO1-Ha1	PV928218
IS-HA/3/10	*S. trutta*	Ddi_CO1-Ha11	PV928955
IS-HA/3/13	*S. trutta*	Ddi_CO1-Ha5	PV928668
IS-HA/3/25	*S. trutta*	Ddi_CO1-Ha1	PV928219
IS-HA/3/32	*S. trutta*	Ddi_CO1-Ha3	PV928300
IS-HA/4/6	*S. trutta*	Ddi_CO1-Ha28	PV929048
IS-HA/4/8	*S. trutta*	Ddi_CO1-Ha29	PV929049
IS-HA/4/13	*S. trutta*	Ddi_CO1-Ha1	PV928220
IS-HA/4/15	*S. trutta*	Ddi_CO1-Ha30	PV929050
IS-HA/5/2	*S. trutta*	**Ddi_CO1-Ha31**	PV929051
IS-HA/5/3	*S. trutta*	Ddi_CO1-Ha2	PV928263
IS-HA/6/7	*S. trutta*	Ddi_CO1-Ha14	PV928998
IS-HA/8/4	*Salvelinus alpinus*	Ddi_CO1-Ha1	PV928221
IS-HA/8/5	*S. alpinus*	Ddi_CO1-Ha3	PV928301
IS-HA/8/7	*S. alpinus*	Ddi_CO1-Ha32	PV929052
IS-HA/8/10	*S. alpinus*	Ddi_CO1-Ha13	PV928995
IS-HA/8/13	*S. alpinus*	Ddi_CO1-Ha33	PV929053
IS-HA/8/15	*S. alpinus*	Ddi_CO1-Ha3	PV928302
IS-HA/8/20	*S. alpinus*	Ddi_CO1-Ha34	PV929054
IS-HA/8/21	*S. alpinus*	Ddi_CO1-Ha3	PV928303
IS-HA/8/23	*S. alpinus*	Ddi_CO1-Ha24	PV929022
IS-HA/8/24	*S. alpinus*	Ddi_CO1-Ha1	PV928222
IS-HA/8/26	*S. alpinus*	Ddi_CO1-Ha3	PV928304
IS-HA/8/38	*S. alpinus*	Ddi_CO1-Ha11	PV928956
IS-HA/8/40	*S. alpinus*	Ddi_CO1-Ha1	PV928223
IS-HA/8/43	*S. alpinus*	Ddi_CO1-Ha1	PV928224
IS-HA/8/61	*S. alpinus*	Ddi_CO1-Ha1	PV928225
IS-HA/9/4	*S. alpinus*	Ddi_CO1-Ha14	PV928999
IS-HA/9/6	*S. alpinus*	Ddi_CO1-Ha22	PV929018
IS-HA/9/7	*S. alpinus*	Ddi_CO1-Ha9	PV928947
IS-HA/9/9	*S. alpinus*	Ddi_CO1-Ha1	PV928226
IS-HA/9/10	*S. alpinus*	Ddi_CO1-Ha14	PV929000
IS-HA/9/12	*S. alpinus*	Ddi_CO1-Ha22	PV929019
IS-HA/9/13	*S. alpinus*	Ddi_CO1-Ha10	PV928951
IS-HA/9/20	*S. alpinus*	Ddi_CO1-Ha1	PV928227
IS-HA/9/23	*S. alpinus*	**Ddi_CO1-Ha8**	PV928943
IS-HA/9/25	*S. alpinus*	Ddi_CO1-Ha11	PV928957
IS-HA/9/31	*S. alpinus*	**Ddi_CO1-Ha12**	PV928992
IS-HA/9/32	*S. alpinus*	Ddi_CO1-Ha21	PV929017
IS-HA/9/35	*S. alpinus*	Ddi_CO1-Ha11	PV928958
**∑ *D. ditremus*: 46**<br>**∑ fish: 8**		**∑ Ddi_CO1-Ha: 1, 2, 3, 5, 7, 8, 9, 10, 11, 12, 13, 14, 21, 22, 24, 28, 29, 30, 31, 32, 33, 34**	

**THINGVALLAVATN (IS-TH)**			
IS-TH/2/16	*S. alpinus*	Ddi_CO1-Ha16	PV929004
IS-TH/2/17	*S. alpinus*	Ddi_CO1-Ha3	PV928308
IS-TH/2/30	*S. alpinus*	Ddi_CO1-Ha4	PV928537
IS-TH/2/46	*S. alpinus*	Ddi_CO1-Ha46	PV929066
IS-TH/3/8	*S. alpinus*	Ddi_CO1-Ha10	PV928954
IS-TH/3/14	*S. alpinus*	Ddi_CO1-Ha23	PV929021
IS-TH/3/23	*S. alpinus*	Ddi_CO1-Ha13	PV928997
IS-TH/5/4	*S. alpinus*	Ddi_CO1-Ha5	PV928670
IS-TH/5/8	*S. alpinus*	Ddi_CO1-Ha1	PV928235
IS-TH/5/12	*S. alpinus*	Ddi_CO1-Ha4	PV928538
IS-TH/5/94	*S. alpinus*	Ddi_CO1-Ha1	PV928236
IS-TH/6/5	*S. alpinus*	Ddi_CO1-Ha47	PV929067
IS-TH/6/14	*S. alpinus*	**Ddi_CO1-Ha48**	PV929068
IS-TH/6/36	*S. alpinus*	Ddi_CO1-Ha49	PV929069
IS-TH/6/48	*S. alpinus*	Ddi_CO1-Ha27	PV929028
IS-TH/10/8	*S. alpinus*	Ddi_CO1-Ha5	PV928671
IS-TH/10/25	*S. alpinus*	Ddi_CO1-Ha50	PV929070
IS-TH/10/33	*S. alpinus*	Ddi_CO1-Ha51	PV929071
IS-TH/10/49	*S. alpinus*	Ddi_CO1-Ha1	PV928237
IS-TH/11/30	*S. alpinus*	Ddi_CO1-Ha52	PV929072
IS-TH/11/38	*S. alpinus*	Ddi_CO1-Ha53	PV929073
IS-TH/11/43	*S. alpinus*	Ddi_CO1-Ha5	PV928672
IS-TH/11/64	*S. alpinus*	Ddi_CO1-Ha4	PV928539
IS-TH/12/9	*S. alpinus*	Ddi_CO1-Ha4	PV928540
IS-TH/12/25	*S. alpinus*	**Ddi_CO1-Ha17**	PV929008
IS-TH/12/52	*S. alpinus*	Ddi_CO1-Ha54	PV929074
IS-TH/12/68	*S. alpinus*	Ddi_CO1-Ha55	PV929075
IS-TH/12/85	*S. alpinus*	Ddi_CO1-Ha1	PV928238
IS-TH/12/94	*S. alpinus*	Ddi_CO1-Ha5	PV928673
IS-TH/15/1	*S. alpinus*	Ddi_CO1-Ha5	PV928674
IS-TH/15/7	*S. alpinus*	Ddi_CO1-Ha5	PV928675
IS-TH/15/10	*S. alpinus*	Ddi_CO1-Ha4	PV928541
IS-TH/15/29	*S. alpinus*	**Ddi_CO1-Ha56**	PV929076
IS-TH/15/36	*S. alpinus*	**Ddi_CO1-Ha57**	PV929077
IS-TH/16/1	*S. alpinus*	Ddi_CO1-Ha27	PV929029
IS-TH/16/6	*S. alpinus*	**Ddi_CO1-Ha17**	PV929009
IS-TH/16/42	*S. alpinus*	Ddi_CO1-Ha4	PV928542
IS-TH/16/50	*S. alpinus*	Ddi_CO1-Ha1	PV928239
IS-TH/18/18	*S. alpinus*	Ddi_CO1-Ha9	PV928950
IS-TH/18/32	*S. alpinus*	Ddi_CO1-Ha4	PV928543
IS-TH/23/5	*S. alpinus*	Ddi_CO1-Ha16	PV929005
IS-TH/23/30	*S. alpinus*	Ddi_CO1-Ha16	PV929006
IS-TH/23/56	*S. alpinus*	Ddi_CO1-Ha58	PV929078
IS-TH/24/3	*S. alpinus*	Ddi_CO1-Ha1	PV928240
IS-TH/24/21	*S. alpinus*	Ddi_CO1-Ha4	PV928544
IS-TH/24/49	*S. alpinus*	Ddi_CO1-Ha1	PV928241
IS-TH/24/76	*S. alpinus*	Ddi_CO1-Ha4	PV928545
**∑ *D. ditremus*: 47**<br>**∑ fish: 12**		**∑ Ddi_CO1-Ha: 1, 3, 4, 5, 9, 10, 13, 16, 17, 23, 27, 46, 47, 48, 49, 50, 51, 52, 53, 54, 55, 56, 57, 58**	

**MÁSVATN (IS-MA)**			
IS-MA/1/4	*S. trutta*	**Ddi_CO1-Ha8**	PV928944
IS-MA/1/5	*S. trutta*	Ddi_CO1-Ha2	PV928264
IS-MA/1/6	*S. trutta*	Ddi_CO1-Ha35	PV929055
IS-MA/1/7	*S. trutta*	Ddi_CO1-Ha2	PV928265
IS-MA/1/8	*S. trutta*	Ddi_CO1-Ha5	PV928669
IS-MA/1/9	*S. trutta*	Ddi_CO1-Ha1	PV928228
IS-MA/1/10	*S. trutta*	Ddi_CO1-Ha2	PV928266
IS-MA/1/11	*S. trutta*	Ddi_CO1-Ha7	PV928744
IS-MA/1/12	*S. trutta*	Ddi_CO1-Ha1	PV928229
IS-MA/1/13	*S. trutta*	Ddi_CO1-Ha10	PV928952
IS-MA/2/1	*S. trutta*	Ddi_CO1-Ha9	PV928948
IS-MA/2/2	*S. trutta*	**Ddi_CO1-Ha36**	PV929056
IS-MA/2/3	*S. trutta*	Ddi_CO1-Ha25	PV929024
IS-MA/3/6	*S. trutta*	Ddi_CO1-Ha37	PV929057
IS-MA/3/7	*S. trutta*	**Ddi_CO1-Ha8**	PV928945
IS-MA/3/8	*S. trutta*	Ddi_CO1-Ha38	PV929058
IS-MA/3/9	*S. trutta*	Ddi_CO1-Ha3	PV928305
IS-MA/3/10	*S. trutta*	Ddi_CO1-Ha7	PV928745
IS-MA/3/11	*S. trutta*	Ddi_CO1-Ha24	PV929023
IS-MA/3/12	*S. trutta*	Ddi_CO1-Ha1	PV928230
IS-MA/3/14	*S. trutta*	Ddi_CO1-Ha1	PV928231
IS-MA/3/15	*S. trutta*	Ddi_CO1-Ha2	PV928267
IS-MA/3/17	*S. trutta*	**Ddi_CO1-Ha18**	PV929010
IS-MA/4/7	*S. trutta*	Ddi_CO1-Ha2	PV928268
IS-MA/4/8	*S. trutta*	Ddi_CO1-Ha1	PV928232
IS-MA/4/9	*S. trutta*	Ddi_CO1-Ha23	PV929020
IS-MA/4/10	*S. trutta*	Ddi_CO1-Ha39	PV929059
IS-MA/4/11	*S. trutta*	Ddi_CO1-Ha3	PV928306
IS-MA/4/12	*S. trutta*	Ddi_CO1-Ha2	PV928269
IS-MA/4/13	*S. trutta*	Ddi_CO1-Ha2	PV928270
IS-MA/4/14	*S. trutta*	Ddi_CO1-Ha13	PV928996
IS-MA/4/15	*S. trutta*	Ddi_CO1-Ha1	PV928233
IS-MA/4/16	*S. trutta*	Ddi_CO1-Ha3	PV928307
IS-MA/4/17	*S. trutta*	Ddi_CO1-Ha40	PV929060
IS-MA/4/21	*S. trutta*	Ddi_CO1-Ha41	PV929061
IS-MA/4/22	*S. trutta*	Ddi_CO1-Ha20	PV929014
IS-MA/4/23	*S. trutta*	Ddi_CO1-Ha26	PV929026
IS-MA/5/9	*S. trutta*	Ddi_CO1-Ha25	PV929025
IS-MA/5/11	*S. trutta*	Ddi_CO1-Ha42	PV929062
IS-MA/5/12	*S. trutta*	Ddi_CO1-Ha10	PV928953
IS-MA/5/13	*S. trutta*	Ddi_CO1-Ha43	PV929063
IS-MA/5/14	*S. trutta*	Ddi_CO1-Ha1	PV928234
IS-MA/5/15	*S. trutta*	Ddi_CO1-Ha2	PV928271
IS-MA/5/16	*S. trutta*	Ddi_CO1-Ha44	PV929064
IS-MA/6/9	*S. trutta*	Ddi_CO1-Ha2	PV928272
IS-MA/6/10	*S. trutta*	Ddi_CO1-Ha2	PV928273
IS-MA/7/9	*S. trutta*	Ddi_CO1-Ha20	PV929015
IS-MA/7/10	*S. trutta*	**Ddi_CO1-Ha45**	PV929065
IS-MA/7/12	*S. trutta*	Ddi_CO1-Ha26	PV929027
IS-MA/8/4	*S. trutta*	Ddi_CO1-Ha9	PV928949
**∑ *D. ditremus*: 50**<br>**∑ fish: 8**		**∑ Ddi_CO1-Ha: 1, 2, 3, 5, 7, 8, 9, 10, 13, 18, 20, 23, 24, 25, 26, 35, 36, 37, 38, 39, 40, 41, 42, 43, 44, 45**	

YTRA-HÓLAVATN (IS-YT)			
IS-YT/1/8	*S. alpinus*	Ddi_CO1-Ha1	PV928242
IS-YT/1/13	*S. alpinus*	**Ddi_CO1-Ha18**	PV929011
IS-YT/1/50	*S. alpinus*	Ddi_CO1-Ha6	PV928715
IS-YT/1/61	*S. alpinus*	Ddi_CO1-Ha2	PV928274
IS-YT/2/1	*S. alpinus*	Ddi_CO1-Ha1	PV928243
IS-YT/2/8	*S. alpinus*	Ddi_CO1-Ha1	PV928244
IS-YT/2/14	*S. alpinus*	**Ddi_CO1-Ha19**	PV929012
IS-YT/2/25	*S. alpinus*	Ddi_CO1-Ha6	PV928716
IS-YT/2/32	*S. alpinus*	**Ddi_CO1-Ha59**	PV929079
IS-YT/3/9	*S. alpinus*	Ddi_CO1-Ha6	PV928717
IS-YT/3/10	*S. alpinus*	Ddi_CO1-Ha6	PV928718
IS-YT/3/20	*S. alpinus*	Ddi_CO1-Ha1	PV928245
IS-YT/3/30	*S. alpinus*	Ddi_CO1-Ha6	PV928719
IS-YT/4/5	*S. alpinus*	Ddi_CO1-Ha1	PV928246
IS-YT/4/8	*S. alpinus*	Ddi_CO1-Ha7	PV928746
IS-YT/4/18	*S. alpinus*	Ddi_CO1-Ha1	PV928247
IS-YT/4/29	*S. alpinus*	**Ddi_CO1-Ha19**	PV929013
IS-YT/4/37	*S. alpinus*	Ddi_CO1-Ha1	PV928248
IS-YT/5/5	*S. alpinus*	Ddi_CO1-Ha15	PV929001
IS-YT/5/6	*S. alpinus*	Ddi_CO1-Ha1	PV928249
IS-YT/5/8	*S. alpinus*	Ddi_CO1-Ha1	PV928250
IS-YT/5/22	*S. alpinus*	Ddi_CO1-Ha15	PV929002
IS-YT/5/30	*S. alpinus*	Ddi_CO1-Ha1	PV928251
IS-YT/6/2	*S. alpinus*	Ddi_CO1-Ha1	PV928252
IS-YT/6/3	*S. alpinus*	Ddi_CO1-Ha60	PV929080
IS-YT/6/4	*S. alpinus*	Ddi_CO1-Ha2	PV928275
IS-YT/6/5	*S. alpinus*	Ddi_CO1-Ha3	PV928309
IS-YT/6/6	*S. alpinus*	Ddi_CO1-Ha1	PV928253
IS-YT/7/3	*S. alpinus*	Ddi_CO1-Ha1	PV928254
IS-YT/8/3	*S. alpinus*	Ddi_CO1-Ha1	PV928255
IS-YT/8/4	*S. alpinus*	Ddi_CO1-Ha1	PV928256
IS-YT/8/5	*S. alpinus*	Ddi_CO1-Ha2	PV928276
IS-YT/9/1	*S. alpinus*	**Ddi_CO1-Ha61**	PV929081
IS-YT/9/4	*S. alpinus*	Ddi_CO1-Ha6	PV928720
IS-YT/9/6	*S. alpinus*	Ddi_CO1-Ha2	PV928277
IS-YT/9/8	*S. alpinus*	Ddi_CO1-Ha7	PV928747
IS-YT/9/9	*S. alpinus*	**Ddi_CO1-Ha62**	PV929082
IS-YT/10/1	*S. alpinus*	Ddi_CO1-Ha15	PV929003
IS-YT/10/3	*S. alpinus*	**Ddi_CO1-Ha12**	PV928993
IS-YT/10/7	*S. alpinus*	Ddi_CO1-Ha1	PV928257
IS-YT/10/8	*S. alpinus*	Ddi_CO1-Ha6	PV928721
IS-YT/10/10	*S. alpinus*	**Ddi_CO1-Ha12**	PV928994
IS-YT/10/11	*S. alpinus*	Ddi_CO1-Ha63	PV929083
IS-YT/13/1	*S. alpinus*	Ddi_CO1-Ha2	PV928278
IS-YT/13/3	*S. alpinus*	Ddi_CO1-Ha1	PV928258
IS-YT/13/5	*S. alpinus*	**Ddi_CO1-Ha8**	PV928946
IS-YT/13/7	*S. alpinus*	Ddi_CO1-Ha64	PV929084
IS-YT/13/10	*S. alpinus*	Ddi_CO1-Ha65	PV929085
IS-YT/13/12	*S. alpinus*	Ddi_CO1-Ha66	PV929086
**∑ *D. ditremus*: 49**<br>**∑ fish: 11**		**∑ Ddi_CO1-Ha: 1, 2, 3, 6, 7, 8, 12, 15, 18, 19, 59, 60, 61, 62, 63, 64, 65, 66**	

Sample codes: the first number indicates the number of the fish; the second number indicates the number of the tapeworm. **Haplotypes in bold and red: haplotypes from distant cluster no. 3.**
