# Supplementary material for: Dibothriocephalosis in salmonids from Iceland: A more complex taxonomic problem than assumed until now?
Source: Curr Res Parasitol Vector Borne Dis. 2025 Aug 30;8:100314. doi: 10.1016/j.crpvbd.2025.100314 (PMC12863047; doi:10.1016/j.crpvbd.2025.100314)
Supplement: Multimedia component 2 [file mmc2.pdf]

Supplementary Table S2. Summary of *Dibothriocephalus dendriticus* specimens from Iceland analysed in the present study and details of mitochondrial cytochrome *c* oxidase subunit 1 haplotypes (Dde_CO1-Ha; 891 bp).

Lake	Host	Dde_CO1-Ha	GenBank Acc. No.
**HAFRAVATN (IS-HA)**			
IS-HA/1/1	*Salmo trutta*	Dde_CO1-Ha1	PV918954
IS-HA/1/2	*S. trutta*	Dde_CO1-Ha2	PV919004
IS-HA/1/3	*S. trutta*	Dde_CO1-Ha1	PV918955
IS-HA/1/4	*S. trutta*	Dde_CO1-Ha11	PV919515
IS-HA/1/8	*S. trutta*	Dde_CO1-Ha1	PV918956
IS-HA/2/3	*S. trutta*	Dde_CO1-Ha2	PV919005
IS-HA/2/4	*S. trutta*	Dde_CO1-Ha1	PV918957
IS-HA/2/8	*S. trutta*	Dde_CO1-Ha2	PV919006
IS-HA/2/10	*S. trutta*	Dde_CO1-Ha4	PV919063
IS-HA/3/1	*S. trutta*	Dde_CO1-Ha3	PV919038
IS-HA/3/18	*S. trutta*	Dde_CO1-Ha5	PV919135
IS-HA/3/20	*S. trutta*	Dde_CO1-Ha5	PV919136
IS-HA/3/26	*S. trutta*	Dde_CO1-Ha10	PV919509
IS-HA/3/34	*S. trutta*	Dde_CO1-Ha4	PV919064
IS-HA/3/38	*S. trutta*	Dde_CO1-Ha3	PV919039
IS-HA/4/7	*S. trutta*	Dde_CO1-Ha2	PV919007
IS-HA/4/14	*S. trutta*	Dde_CO1-Ha1	PV918958
IS-HA/5/1	*S. trutta*	Dde_CO1-Ha1	PV918959
IS-HA/5/4	*S. trutta*	Dde_CO1-Ha4	PV919065
IS-HA/5/5	*S. trutta*	Dde_CO1-Ha3	PV919040
IS-HA/5/6	*S. trutta*	Dde_CO1-Ha3	PV919041
IS-HA/6/1	*S. trutta*	Dde_CO1-Ha3	PV919042
IS-HA/6/11	*S. trutta*	Dde_CO1-Ha2	PV919008
IS-HA/8/2	*Salvelinus alpinus*	Dde_CO1-Ha10	PV919510
IS-HA/9/1	*S. alpinus*	Dde_CO1-Ha1	PV918960
IS-HA/9/2	*S. alpinus*	Dde_CO1-Ha1	PV918961
IS-HA/9/3	*S. alpinus*	Dde_CO1-Ha2	PV919009
**∑ *D. dendriticus*: 27**<br>**∑ fish: 8**		**∑ Dde_CO1-Ha: 1, 2, 3, 4, 5, 10, 11**	

THINGVALLAVATN (IS-TH)			
IS-TH/2/1	*S. alpinus*	Dde_CO1-Ha3	PV919052
IS-TH/2/2	*S. alpinus*	Dde_CO1-Ha3	PV919053
IS-TH/2/14	*S. alpinus*	Dde_CO1-Ha3	PV919054
IS-TH/2/24	*S. alpinus*	Dde_CO1-Ha1	PV918972
IS-TH/2/32	*S. alpinus*	Dde_CO1-Ha2	PV919025
IS-TH/2/33	*S. alpinus*	Dde_CO1-Ha4	PV919066
IS-TH/2/34	*S. alpinus*	Dde_CO1-Ha3	PV919055
IS-TH/3/1	*S. alpinus*	Dde_CO1-Ha2	PV919026
IS-TH/3/4	*S. alpinus*	Dde_CO1-Ha1	PV918973
IS-TH/3/5	*S. alpinus*	Dde_CO1-Ha9	PV919490
IS-TH/3/18	*S. alpinus*	Dde_CO1-Ha1	PV918974
IS-TH/3/33	*S. alpinus*	Dde_CO1-Ha4	PV919067
IS-TH/3/42	*S. alpinus*	Dde_CO1-Ha1	PV918975
IS-TH/5/2	*S. alpinus*	Dde_CO1-Ha3	PV919056
IS-TH/5/14	*S. alpinus*	Dde_CO1-Ha2	PV919027
IS-TH/5/15	*S. alpinus*	Dde_CO1-Ha1	PV918976
IS-TH/5/16	*S. alpinus*	Dde_CO1-Ha8	PV919475
IS-TH/5/24	*S. alpinus*	Dde_CO1-Ha13	PV919517
IS-TH/6/7	*S. alpinus*	Dde_CO1-Ha2	PV919028
IS-TH/6/9	*S. alpinus*	Dde_CO1-Ha1	PV918977
IS-TH/6/10	*S. alpinus*	Dde_CO1-Ha4	PV919068
IS-TH/6/12	*S. alpinus*	Dde_CO1-Ha14	PV919518
IS-TH/10/3	*S. alpinus*	Dde_CO1-Ha2	PV919029
IS-TH/10/9	*S. alpinus*	Dde_CO1-Ha1	PV918978
IS-TH/10/10	*S. alpinus*	Dde_CO1-Ha9	PV919491
IS-TH/11/1	*S. alpinus*	Dde_CO1-Ha3	PV919057
IS-TH/11/2	*S. alpinus*	Dde_CO1-Ha3	PV919058
IS-TH/11/4	*S. alpinus*	Dde_CO1-Ha3	PV919059
IS-TH/11/5	*S. alpinus*	Dde_CO1-Ha1	PV918979
IS-TH/12/1	*S. alpinus*	Dde_CO1-Ha9	PV919492
IS-TH/12/2	*S. alpinus*	Dde_CO1-Ha4	PV919069
IS-TH/12/3	*S. alpinus*	Dde_CO1-Ha3	PV919060
IS-TH/12/4	*S. alpinus*	Dde_CO1-Ha1	PV918980
IS-TH/15/2	*S. alpinus*	Dde_CO1-Ha1	PV918981
IS-TH/15/3	*S. alpinus*	Dde_CO1-Ha1	PV918982
IS-TH/15/4	*S. alpinus*	Dde_CO1-Ha1	PV918983
IS-TH/15/5	*S. alpinus*	Dde_CO1-Ha15	PV919519
IS-TH/16/21	*S. alpinus*	Dde_CO1-Ha1	PV918984
IS-TH/16/37	*S. alpinus*	Dde_CO1-Ha4	PV919070
IS-TH/16/59	*S. alpinus*	Dde_CO1-Ha1	PV918985
IS-TH/18/3	*S. alpinus*	Dde_CO1-Ha1	PV918986
IS-TH/18/4	*S. alpinus*	Dde_CO1-Ha1	PV918987
IS-TH/18/5	*S. alpinus*	Dde_CO1-Ha1	PV918988
IS-TH/23/2	*S. alpinus*	Dde_CO1-Ha3	PV919061
IS-TH/23/3	*S. alpinus*	Dde_CO1-Ha16	PV919520
IS-TH/23/4	*S. alpinus*	Dde_CO1-Ha3	PV919062
IS-TH/24/4	*S. alpinus*	Dde_CO1-Ha1	PV918989
IS-TH/24/9	*S. alpinus*	Dde_CO1-Ha1	PV918990
IS-TH/24/10	*S. alpinus*	Dde_CO1-Ha2	PV919030
IS-TH/24/24	*S. alpinus*	Dde_CO1-Ha2	PV919031
IS-TH/24/32	*S. alpinus*	Dde_CO1-Ha4	PV919071
**∑ *D. dendriticus*: 51**<br>**∑ fish: 12**		**∑ Dde_CO1-Ha: 1, 2, 3, 4, 8, 9, 13, 14, 15, 16**	

**MÁSVATN (IS-MA)**			
IS-MA/1/1	*S. trutta*	Dde_CO1-Ha2	PV919010
IS-MA/1/2	*S. trutta*	Dde_CO1-Ha1	PV918962
IS-MA/1/3	*S. trutta*	Dde_CO1-Ha1	PV918963
IS-MA/3/1	*S. trutta*	Dde_CO1-Ha1	PV918964
IS-MA/3/2	*S. trutta*	Dde_CO1-Ha1	PV918965
IS-MA/3/4	*S. trutta*	Dde_CO1-Ha2	PV919011
IS-MA/3/5	*S. trutta*	Dde_CO1-Ha7	PV919461
IS-MA/4/1	*S. trutta*	Dde_CO1-Ha2	PV919012
IS-MA/4/2	*S. trutta*	Dde_CO1-Ha6	PV919149
IS-MA/4/3	*S. trutta*	Dde_CO1-Ha6	PV919150
IS-MA/4/4	*S. trutta*	Dde_CO1-Ha3	PV919043
IS-MA/4/5	*S. trutta*	Dde_CO1-Ha3	PV919044
IS-MA/4/6	*S. trutta*	Dde_CO1-Ha5	PV919137
IS-MA/4/26	*S. trutta*	Dde_CO1-Ha3	PV919045
IS-MA/5/1	*S. trutta*	Dde_CO1-Ha5	PV919138
IS-MA/5/2	*S. trutta*	Dde_CO1-Ha2	PV919013
IS-MA/5/3	*S. trutta*	Dde_CO1-Ha5	PV919139
IS-MA/5/4	*S. trutta*	Dde_CO1-Ha2	PV919014
IS-MA/5/5	*S. trutta*	Dde_CO1-Ha3	PV919046
IS-MA/5/6	*S. trutta*	Dde_CO1-Ha2	PV919015
IS-MA/5/7	*S. trutta*	Dde_CO1-Ha5	PV919140
IS-MA/5/8	*S. trutta*	Dde_CO1-Ha2	PV919016
IS-MA/5/10	*S. trutta*	Dde_CO1-Ha2	PV919017
IS-MA/6/2	*S. trutta*	Dde_CO1-Ha2	PV919018
IS-MA/6/4	*S. trutta*	Dde_CO1-Ha2	PV919019
IS-MA/6/6	*S. trutta*	Dde_CO1-Ha5	PV919141
IS-MA/6/7	*S. trutta*	Dde_CO1-Ha2	PV919020
IS-MA/7/1	*S. trutta*	Dde_CO1-Ha8	PV919473
IS-MA/7/2	*S. trutta*	Dde_CO1-Ha1	PV918966
IS-MA/7/3	*S. trutta*	Dde_CO1-Ha1	PV918967
IS-MA/7/4	*S. trutta*	Dde_CO1-Ha2	PV919021
IS-MA/7/5	*S. trutta*	Dde_CO1-Ha2	PV919022
IS-MA/7/6	*S. trutta*	Dde_CO1-Ha3	PV919047
IS-MA/7/7	*S. trutta*	Dde_CO1-Ha1	PV918968
IS-MA/7/8	*S. trutta*	Dde_CO1-Ha2	PV919023
IS-MA/8/1	*S. trutta*	Dde_CO1-Ha8	PV919474
IS-MA/8/2	*S. trutta*	Dde_CO1-Ha5	PV919142
IS-MA/9/1	*S. trutta*	Dde_CO1-Ha1	PV918969
IS-MA/9/2	*S. trutta*	Dde_CO1-Ha3	PV919048
IS-MA/9/3	*S. trutta*	Dde_CO1-Ha3	PV919049
IS-MA/9/4	*S. trutta*	Dde_CO1-Ha1	PV918970
IS-MA/9/5	*S. trutta*	Dde_CO1-Ha6	PV919151
IS-MA/10/1	*S. trutta*	Dde_CO1-Ha1	PV918971
IS-MA/10/2	*S. trutta*	Dde_CO1-Ha12	PV919516
IS-MA/10/3	*S. trutta*	Dde_CO1-Ha7	PV919462
IS-MA/10/5	*S. trutta*	Dde_CO1-Ha3	PV919050
IS-MA/10/6	*S. trutta*	Dde_CO1-Ha2	PV919024
IS-MA/10/7	*S. trutta*	Dde_CO1-Ha7	PV919463
IS-MA/11/1	*S. trutta*	Dde_CO1-Ha6	PV919152
IS-MA/11/2	*S. trutta*	Dde_CO1-Ha3	PV919051
**∑ *D. dendriticus*: 50**<br>**∑ fish: 10**		**∑ Dde_CO1-Ha: 1, 2, 3, 5, 6, 7, 8, 12**	

YTRA-HÓLAVATN (IS-YT)			
IS-YT/1/47	*S. alpinus*	Dde_CO1-Ha6	PV919153
IS-YT/5/3	*S. alpinus*	Dde_CO1-Ha2	PV919032
IS-YT/5/4	*S. alpinus*	Dde_CO1-Ha2	PV919033
IS-YT/5/18	*S. alpinus*	Dde_CO1-Ha2	PV919034
**∑ *D. dendriticus*: 4**<br>**∑ fish: 2**		**∑ Dde_CO1-Ha: 2, 6**	

Sample codes: the first number indicates the number of the fish; the second number indicates the number of the tapeworm.
