# Supplementary material for: Dibothriocephalosis in salmonids from Iceland: A more complex taxonomic problem than assumed until now?
Source: Curr Res Parasitol Vector Borne Dis. 2025 Aug 30;8:100314. doi: 10.1016/j.crpvbd.2025.100314 (PMC12863047; doi:10.1016/j.crpvbd.2025.100314)
Supplement: Multimedia component 3 [file mmc3.pdf]

Supplementary Table S3. Summary data on mitochondrial *cox*1 haplotypes (CO1-Ha; 891 bp) of *Dibothriocephalus ditremus* (Ddi) from Iceland.

Haplotype	HA	TH	MA	YT	no. M	M position	ts/tv	s/ns
**Ddi_CO1-Ha1**	+	+	+	+	**The reference haplotype**			
Ddi_CO1-Ha2	+		+	+	1	276	ts	s
Ddi_CO1-Ha3	+	+	+	+	2	243, 276	ts, ts	s
Ddi_CO1-Ha4		+			1	777	ts	s
Ddi_CO1-Ha5	+	+	+		2	126, 552	tv, ts	s
Ddi_CO1-Ha6				+	3	243, 276, ***637***	ts, ts, ts	s, ***ns***
Ddi_CO1-Ha7	+		+	+	1	333	tv	s
**Ddi_CO1-Ha8**	+		+	+	**26**	19, 24, 42, 63, 66, 69, 117, 168, 255, 339, 366, 486, 498, 543, 549, 603, 621, 684, 696, 759, 789, 813, 820, 822, 846, 882	ts, ts, ts, ts, ts, tv, ts, ts, ts, tv, ts, ts, ts, ts, ts, ts, ts, ts, ts, tv, ts, ts, ts, ts, ts, ts	s
Ddi_CO1-Ha9	+	+	+		3	156, 411, 759	ts, ts, ts	s
Ddi_CO1-Ha10	+	+	+		1	366	ts	s
Ddi_CO1-Ha11	+				1	732	ts	s
**Ddi_CO1-Ha12**	+			+	**28**	19, 24, 66, 69, 114, 117, 168, 213, 255, 339, 366, 486, 498, 507, 543, 549, 570, 603, 621, 654, 657, 684, 696, 759, 813, 822, 846, 882	ts, ts, ts, tv, ts, ts, ts, ts, ts, tv, ts, ts, ts, ts, ts, ts, ts, ts, ts, ts, ts, ts, ts, tv, ts, ts, ts, ts	s
Ddi_CO1-Ha13	+	+	+		3	63, 558, 636	tv, ts, ts	s
Ddi_CO1-Ha14	+				6	75, 126, 168, 315, 486, 543	ts, ts, ts, ts, ts, ts	s
Ddi_CO1-Ha15				+	1	690	ts	s
Ddi_CO1-Ha16		+			2	777, ***821***	ts, ts	s, ***ns***
**Ddi_CO1-Ha17**		+			**26**	19, 24, 42, 63, 66, 69, 117, 168, 255, 339, 366, 486, 498, 543, 549, 603, 621, 684, 696, 720, 759, 789, 813, 820, 846, 882	ts, ts, ts, ts, ts, tv, ts, ts, ts, tv, ts, ts, ts, ts, ts, ts, ts, ts, ts, ts, tv, ts, ts, ts, ts, ts	s
**Ddi_CO1-Ha18**			+	+	**26**	19, 24, 42, 63, 66, 69, 117, 168, 255, 339, 366, 486, 498, 543, 549, 621, 684, 696, 720, 759, 789, 813, 820, 822, 846, 882	ts, ts, ts, tv, ts, tv, ts, ts, ts, tv, ts, ts, ts, ts, ts, ts, ts, ts, ts, tv, ts, ts, ts, ts, ts, ts	s
**Ddi_CO1-Ha19**				+	**25**	19, 24, 42, 63, 69, 117, 255, 339, 366, 486, 498, 543, 549, 621, 684, 696, 714, 720, 759, 789, 813, 820, 822, 846, 882	ts, ts, ts, tv, tv, ts, ts, tv, ts, ts, ts, ts, ts, ts, ts, ts, ts, ts, tv, ts, ts, ts, ts, ts, ts	s
Ddi_CO1-Ha20			+		3	108, 243, 276	ts, ts, ts	s
Ddi_CO1-Ha21	+				7	168, 315, 486, 543, 690, 699, 720	ts, ts, ts, ts, ts, ts, ts	s
Ddi_CO1-Ha22	+				6	168, 216, 282, 486, 543, 720	ts, ts, ts, ts, ts, ts	s
Ddi_CO1-Ha23		+	+		1	208	ts	s
Ddi_CO1-Ha24	+		+		3	276, 585, 657	ts, ts, ts	s
Ddi_CO1-Ha25			+		2	372, 777	ts, ts	s
Ddi_CO1-Ha26			+		1	552	ts	s

Ddi_CO1-Ha27		+		1	846	ts	s
Ddi_CO1-Ha28	+			1	687	ts	s
Ddi_CO1-Ha29	+			1	210	ts	s
Ddi_CO1-Ha30	+			4	168, 210, 846, ***857***	ts, ts, ts, tv	s, ***ns***
**Ddi_CO1-Ha31**	+			**34**	19, 24, 42, 66, 69, 117, 168, 189, 207, 213, 255, 261, 280, 339, 486, 492, 498, 501, 543, 549, 570, 594, 603, 684, 696, 720, 723, 741, 759, 813, 819, 822, 846, 882	ts, ts, ts, ts, tv, ts, ts, ts, ts, ts, ts, ts, ts, tv, ts, ts, ts, ts, ts, ts, ts, ts, ts, ts, ts, ts, ts, ts, tv, ts, tv, ts, ts, ts	s
Ddi_CO1-Ha32	+			1	588	tv	s
Ddi_CO1-Ha33	+			2	66, 276	ts, ts	s
Ddi_CO1-Ha34	+			8	168, 315, 486, 543, 690, 699, 720, 820	ts, ts, ts, ts, ts, ts, ts, ts	s
Ddi_CO1-Ha35			+	6	168, 282, 486, 543, ***862, 866***	ts, ts, ts, ts, ts, ts	s, ***ns***
**Ddi_CO1-Ha36**			+	**27**	19, 24, 66, 69, 117, 168, 213, 255, 339, 366, 453, 486, 498, 507, 543, 549, 570, 603, 621, 654, 657, 684, 696, 759, 813, 822, 846	ts, ts, ts, tv, ts, ts, ts, ts, tv, ts, ts, ts, ts, ts, ts, ts, ts, ts, ts, ts, ts, ts, ts, tv, ts, ts, ts	s
Ddi_CO1-Ha37			+	7	168, 315, 486, 543, 633, 699, 720	ts, ts, ts, ts, ts, ts, ts	s
Ddi_CO1-Ha38			+	6	168, 315, 486, 543, 654, 720	ts, ts, ts, ts, ts, ts	s
**Ddi_CO1-Ha39**			+	**26**	19, 24, 42, 63, 66, 69, 117, 168, 255, 339, 366, 486, 498, 543, 549, 621, 684, 696, 759, ***778***, 789, 813, 820, 822, 846, 882	ts, ts, ts, ts, ts, tv, ts, ts, ts, tv, ts, ts, ts, ts, ts, ts, ts, ts, tv, tv, ts, ts, ts, ts, ts, ts	s, ***ns***
Ddi_CO1-Ha40			+	1	***386***	ts	***ns***
Ddi_CO1-Ha41			+	1	***148***	tv	***ns***
Ddi_CO1-Ha42			+	2	211, 411	ts, ts	s
Ddi_CO1-Ha43			+	5	168, 315, 486, 543, 720	ts, ts, ts, ts, ts	s
Ddi_CO1-Ha44			+	1	***383***	tv	***ns***
**Ddi_CO1-Ha45**			+	**27**	19, 24, 66, 69, 117, 168, 213, 255, 339, 366, 486, 498, 507, 543, 549, 570, 603, 621, 654, 657, 684, 696, 759, 813, 822, 846, 882	ts, ts, ts, tv, ts, ts, ts, ts, tv, ts, ts, ts, ts, ts, ts, ts, ts, ts, ts, ts, ts, ts, tv, ts, ts, ts, ts	s
Ddi_CO1-Ha46		+		1	606	ts	s
Ddi_CO1-Ha47		+		2	36, 777	ts, ts	s
**Ddi_CO1-Ha48**		+		**25**	19, 24, 42, 63, 66, 69, 117, 168, 255, 339, 366, 486, 498, 543, 549, 621, 684, 696, 759, 789, 813, 820, 822, 846, 882	ts, ts, ts, ts, ts, tv, ts, ts, ts, tv, ts, ts, ts, ts, ts, ts, ts, ts, tv, ts, ts, ts, ts, ts, ts	s
Ddi_CO1-Ha49		+		2	246, 756	ts, ts	s
Ddi_CO1-Ha50		+		7	168, 315, 486, 543, 699, 720, 810	ts, ts, ts, ts, ts, ts, ts	s
Ddi_CO1-Ha51		+		2	24, 81	ts, ts	s
Ddi_CO1-Ha52		+		1	342	ts	s
Ddi_CO1-Ha53		+		3	16, 777, 792	ts, ts, ts	s
Ddi_CO1-Ha54		+		2	156, 411	ts, ts	s

Ddi_CO1-Ha55	+				2	285, 690	ts, tv	s
**Ddi_CO1-Ha56**	+				**27**	19, 24, 42, 63, 66, 69, 117, 168, 255, 339, 366, 486, 498, 543, 549, 603, 621, 684, 696, 759, 789, 813, 820, 822, 846, 855, 882	ts, ts, ts, ts, ts, tv, ts, ts, ts, tv, ts, ts, ts, ts, ts, ts, ts, ts, ts, tv, ts, ts, ts, ts, ts, ts, ts	s
**Ddi_CO1-Ha57**	+				**27**	19, 24, 42, ***59***, 63, 66, 69, 117, 168, 255, 339, 366, 486, 498, 543, 549, 603, 621, 684, 696, 720, 759, 789, 813, 820, 846, 882	ts, ts, ts, ts, ts, ts, tv, ts, ts, ts, tv, ts, ts, ts, ts, ts, ts, ts, ts, ts, ts, tv, ts, ts, ts, ts, ts	s, ***ns***
Ddi_CO1-Ha58	+				1	792	ts	s
**Ddi_CO1-Ha59**				+	**25**	19, 42, 63, 66, 69, 117, 168, 255, 339, 366, ***389***, 486, 498, 543, 549, 621, 684, 696, 759, 789, 813, 820, 822, 846, 882	ts, ts, ts, ts, tv, ts, ts, ts, tv, ts, ts, ts, ts, ts, ts, ts, ts, ts, tv, ts, ts, ts, ts, ts, ts	s, ***ns***
Ddi_CO1-Ha60				+	5	168, 315, ***415***, 486, 543	ts, ts, ts, ts, ts	s, ***ns***
**Ddi_CO1-Ha61**				+	**26**	19, 24, 42, 63, 66, 69, 117, 168, 255, 258, 339, 366, 486, 498, 543, 549, 621, 684, 696, 759, 789, 813, 820, 822, 846, 882	ts, ts, ts, ts, ts, tv, ts, ts, ts, ts, tv, ts, ts, ts, ts, ts, ts, ts, ts, tv, ts, ts, ts, ts, ts, ts	s
**Ddi_CO1-Ha62**				+	**26**	19, 24, 42, 63, 66, 69, 117, 168, 255, 339, 366, 486, 498, 543, 549, 621, 642, 684, 696, 759, 789, 813, 820, 822, 846, 882	ts, ts, ts, ts, ts, tv, ts, ts, ts, tv, ts, ts, ts, ts, ts, ts, ts, ts, ts, tv, ts, ts, ts, ts, ts, ts	s
Ddi_CO1-Ha63				+	8	24, 168, 315, 486, 543, 633, 699, 720	ts, ts, ts, ts, ts, ts, ts, ts	s
Ddi_CO1-Ha64				+	7	16, 168, 282, 361, 486, 543, 720	ts, ts, ts, ts, ts, ts, ts	s
Ddi_CO1-Ha65				+	1	69	ts	s
Ddi_CO1-Ha66				+	6	168, 315, 486, 543, 720, 841	ts, ts, ts, ts, ts, ts	s

HA, Hafravatn; **TH**, Thingvallavatn; **MA**, Másvatn; **YT**, Ytra-Hólavatn; **+**, haplotype detected in the lake; **no. M**, number of mutations in respect to the reference haplotype CO1-Ha1; **M**, mutation; **ts**, transition; **tv**, transversion; **s**, synonymous mutation; **ns**, nonsynonymous mutation.

- Positions of mutations are numbered within the amplified region of *cox*1 gene (891 bp) and not within the complete *cox*1 gene (1566 bp).
- Mutations in ***bold and italics*** correspond to the ***nonsynonymous*** mutations.
- Haplotypes in **bold and red letters** and highlighted in yellow fields are haplotypes from the distinct cluster no. 3.
- The most distant haplotype Ha31 is highlighted in blue field.
