# Supplementary material for: Dibothriocephalosis in salmonids from Iceland: A more complex taxonomic problem than assumed until now?
Source: Curr Res Parasitol Vector Borne Dis. 2025 Aug 30;8:100314. doi: 10.1016/j.crpvbd.2025.100314 (PMC12863047; doi:10.1016/j.crpvbd.2025.100314)
Supplement: Multimedia component 4 [file mmc4.pdf]

Supplementary Table S4. Summary data on mitochondrial *cox*1 haplotypes (CO1-Ha; 891 bp) of *Dibothriocephalus dendriticus* (Dde) from Iceland.

Haplotype	HA	TH	MA	YT	no. M	M position	ts/tv	s/ns
**Dde_CO1-Ha1**	+	+	+		**The reference haplotype**			
Dde_CO1-Ha2	+	+	+	+	7	147, 357, 360, 366, 405, 459, 855	ts, tv, ts, ts, ts, ts, ts	s
Dde_CO1-Ha3	+	+	+		2	234, 372	ts, ts	s
Dde_CO1-Ha4	+	+			6	243, 357, 366, 405, 459, 855	ts, tv, ts, ts, ts, ts	s
Dde_CO1-Ha5	+		+		3	234, 372, 624	ts, ts, ts	s
Dde_CO1-Ha6			+	+	3	79, 234, 372	ts, ts, ts	s
Dde_CO1-Ha7			+		3	114, 372, 552	ts, ts, ts	s
Dde_CO1-Ha8		+	+		3	***150***, 234, 372	ts, ts, ts	***ns***, s
Dde_CO1-Ha9		+			3	234, 372, 546	ts, ts, ts	s
Dde_CO1-Ha10	+				1	600	ts	s
Dde_CO1-Ha11	+				6	357, 360, 366, 405, 459, 855	tv, ts, ts, ts, ts, ts	s
Dde_CO1-Ha12			+		8	243, 357, 366, 405, 459, 507, ***760***, 855	ts, ts, ts, ts, ts, ts, ts, ts	s, ***ns***
Dde_CO1-Ha13		+			4	225, 459, 622, ***647***	ts, ts, ts, ts	s, ***ns***
Dde_CO1-Ha14		+			1	510	ts	s
Dde_CO1-Ha15		+			1	***761***	ts	***ns***
Dde_CO1-Ha16		+			8	147, 357, 360, 366, 405, 459, 690, 855	ts, tv, ts, ts, ts, ts, ts, ts	s

HA, Hafravatn; **TH**, Thingvallavatn; **MA**, Másvatn; **YT**, Ytra-Hólavatn; **+**, haplotype detected in the lake; **no. M**, number of mutations in respect to the reference haplotype CO1-Ha1; **M**, mutation; **ts**, transition; **tv**, transversion; **s**, synonymous mutation; **ns**, nonsynonymous mutation.

• Positions of mutations are numbered within the amplified region of *cox*1 gene (891 bp) and not within the complete *cox*1 gene (1566 bp).

• Mutations in ***bold and italics*** correspond to the nonsynonymous mutations.
